# Study of Carbon Black Types in SBR Rubber: Mechanical and Vibration Damping Properties

**DOI:** 10.3390/ma13102394

**Published:** 2020-05-22

**Authors:** Marek Pöschl, Martin Vašina, Petr Zádrapa, Dagmar Měřínská, Milan Žaludek

**Affiliations:** 1 Centre of Polymer Systems, University Institute, Tomas Bata University in Zlin, Třída Tomáše Bati 5678, 760 01 Zlin, Czech Republic; poschl@utb.cz (M.P.); zadrapa@utb.cz (P.Z.); 2 Faculty of Technology, Tomas Bata University in Zlin, Vavrečkova 275, 760 01 Zlin, Czech Republic; merinska@utb.cz (D.M.); zaludek@utb.cz (M.Ž.); 3 Faculty of Mechanical Engineering, VŠB-Technical University of Ostrava, 17. Listopadu 15/2172, 708 33 Ostrava-Poruba, Czech Republic

**Keywords:** vulcanized rubber, rubber compounds, carbon black, vibration damping, viscoelasticity

## Abstract

Styrene–butadiene rubber mixtures with four types of carbon black were studied in this paper. The mechanical properties, including the ability to damp mechanical vibration, were investigated, along with dynamical mechanical analysis (DMA). It has been found that carbon black types N 110 and N 330, having the largest specific surface area and the smallest particle diameter, provide a good stiffening effect. These particles have significant interactions between the rubber, resulting in good reinforcement. On the other hand, the carbon black N 990 type has a lower reinforcing effect and improved vibration damping properties at higher excitation frequencies due to higher dissipation of mechanical energy into heat under dynamic loading. The effect of the number of loading cycles on vibration damping properties of the rubber composites was also investigated in this study. It can be concluded that the abovementioned properties of the investigated rubber composites correspond to physical–mechanical properties of the applied carbon black types.

## 1. Introduction

Mechanical vibration, which is caused by oscillation of a mechanical or structural system about an equilibrium position, is an undesirable phenomenon in many cases (e.g., manufacturing processes, means of transport, and in home appliances). Furthermore, the mechanical vibration can contribute to excessive noise and have a negative effect on labor protection, manufacturing quality, and productivity. For these reasons, it is necessary to eliminate the mechanical vibration through appropriate measures, e.g., by application of suitable vibration damping materials [1,2,3].

Polymers have become more frequently used in a variety of applications in order to diminish vibration [2] in cars, ships, and other products where machinery creates vibration [4] or there are natural causes of vibration, which should be lowered from the environment in question. Anechoic chambers exist that allow for accurate observation of sound energy. Given or created energy is treated by dissipation or absorption by a variety of mechanisms [5].

In an engineering system, a structure should stay stable and undamaged despite internal and external vibrations. The stability of a system depends on its damping ability, which can be influenced by the composition of rubber mixtures [6,7]. Damping is defined as the energy dissipation of a vibration system and it can be presented by various parameters, one of which is the loss factor. The loss factor can be determined by several different methods, which are grouped into time, deformation, temperature, and frequency sweep tests [7,8].

All rubber mixture’s properties are mainly influenced by its composition. The rubber type is only the base of the compound. In order to achieve the best properties in the finished rubber product, it is necessary to add some additives to the rubber to significantly change the properties of the product and improve its resistance to various undesirable effects. The additive component is given in units of phr, which is the weight per 100 parts by weight of rubber [9]. The most important rubber components include the vulcanizing agents, accelerators, activators, retarders (inhibitors), antidegradants, fillers, plasticizers, and special additives [10,11,12,13].

Two main types of additives are involved in the material’s ability to dampen mechanical vibration. Firstly, the oil or plasticizer significantly contributes to determining the damping characteristics. The oil choice is usually decided by the required low temperature flexibility and by damping characteristics. Secondly, the carbon black type and its amount are significant factors that influence the damping characteristics of a rubber compound [14,15,16].

The aim of this study is to investigate mechanical and vibration damping properties of rubber composites containing different carbon black primary particle sizes with slightly different properties. In order to determine the influence of a carbon black structure on mechanical and vibration damping properties, the rubber mixture compositions were prepared with the same carbon black volume concentration.

## 2. Materials and Methods

### 2.1. Materials

Styrene butadiene rubber (SBR) SBR 1500 (Synthos Kralupy a.s., Kralupy nad Vltavou, Czech Republic), was used as the base for rubber compounds. Carbon black grades N 110, N 330, N 550, and N 990 from CS Cabot, s.r.o. (Valašské Meziříčí, Czech Republic), were used as fillers. The additives, including ZnO from SlovZink a.s. (Košeca, Slovakia), stearic acid from Setuza a.s. (Ústí nad Labem, Czech Republic), N-tert-butyl-benzothiazole sulfonamide (TBBS) from Duslo a.s. (Šaľa, Slovakia), and sulphur type Crystex OT33 from Eastman Chemical company (Kingsport, Tennessee, USA), were also compounded. The rubber recipe is given in Table 1.

Iodine adsorption (IA) and dibutyl phthalate absorption (DBPA) methods were used in order to characterize the carbon black more precisely. While the surface area could be characterized by the first method, the second one was used for secondary structure characterization. The IA of the studied carbon black types was 145, 82, 43, and 10 g × kg^−1^ for N 110, N 330, N 550, and N 990, respectively. The secondary structure was more similar for N 110, N 330, and N 550 carbon black types (113, 102, and 121 mL × 100 g^−1^, respectively). However, the last filler type (N 990) was characterized by the lowest secondary structure, with a DBPA of 35 mL × 100 g^−1^. The primary carbon black particle sizes were measured using JEM-2100 transmission electron microscope (JEOL Ltd., Tokyo, Japan) and the average diameters of the studied carbon black were about 15 nm for N 110, 27 nm for N 330, 55 nm for N 550, and 280 nm for N 990. The optimal filler concentration in a polymer matrix is strongly influenced by the stiffening effect (particle size, shape, and structure) of the filler. With decreasing particle size, the stiffening effect increases. A filler value of 50 phr was chosen as the optimal concentration for various types of carbon black to be able to evaluate their effects on the studied properties. At the given phr concentration, most of rubber mixtures exhibits optimal physical-mechanical properties.

### 2.2. Obtaining the Mixtures and Sample Preparation

The basic rubber compounds (SBR, carbon black, ZnO, and stearic acid) were mixed in a Banbury laboratory internal mixer (Farrel Pominy, Castellanza, Italy) (volume 0.41 L, fill factor 0.72, chamber temperature 70 °C) for 8 min at 80 rpm. Consequently, the compounds were cooled down to 80 °C over a period of 1 min on a double roll mill (150 mm × 330 mm, Farrel) at a rotation speed ratio of 1:1.2. Finally, the accelerator and sulphur were added and mixed for the next 6 min.

### 2.3. Methods

#### 2.3.1. Curing Characterization

Curing characteristics were measured on the Premier MDR moving die rheometer from Monsanto Company (Dayton, Ohio, USA) at a constant temperature of 160 °C. Scorch (*t_s_*_1_) and optimum cure (*t*_90_) times were evaluated by these measurements.

Consequently, compression molding in a hydraulic press at 20 MPa and 160 °C was used for the test sheet preparation. The samples sheets (150 mm × 150 mm) for mechanical tests measured 1 and 2 mm in thickness, cylinders measured 10, 15, 20, and 80 mm in diameter for vibration tests, and cylinders measured 20 and 100 mm in diameter for cyclic pressure tests. The samples measuring up to 2 mm in thickness were molded over the given time period, which is given by the time *t*_90_. The curing time for samples thicker than 2 mm was calculated as the value of time *t*_90_ with the addition of 1 min per each 1 mm of sample thickness.

#### 2.3.2. Quasistatic Mechanical Properties

Tensile tests were performed on the T10D tensile testing machine from Alpha Technology (Hudson, Ohio, USA), according to ISO 37 standard. Ten specimens (type 2, elongation speed of 500 mm/min) were tested in this case. The average values of the measured quantities and their standard deviations were subsequently evaluated.

Shore A hardness was measured according to ISO 7619 standard. Samples measuring 6 mm in thickness were tested and the parameters (median and arithmetic average) were obtained from nine measured values.

Rubber rebound resilience was measured according to ISO 4662 standard. The sample measuring 10 mm in thickness was examined and the average values of the resilience, including its standard deviations from 6 values, were subsequently determined. These tests were carried out at an ambient temperature of 22 °C.

#### 2.3.3. Dynamical Mechanical Properties

Dynamical mechanical analysis (DMA) of the vulcanized rubber samples measuring *t* = 1 mm in thickness and *w* = 6 mm in width was realized on the Mettler Toledo DMA 1 equipment (Columbus, Ohio, USA) in tensile mode. Firstly, the temperature sweep was measured in a temperature range of −80 °C to +20 °C, with a temperature rate of 2 °C/min, at a constant deformation rate of 5 µm and a frequency of 20 Hz. Secondly, the frequency sweep was studied at a constant deformation rate of 10 µm and at 25 °C, with varying frequency ranging from 0 to 250 Hz. Finally, the strain sweep was studied at a constant frequency of 20 Hz and at 25 °C, with varying strain ranging from 0 to 200 µm. Three measurements were taken for each compound.

The dynamic shear properties were measured on the Premier RPA rubber process analyzer from Alpha Technology (Hudson, Ohio, USA). Rubber compounds were firstly cured for *t*_90_ at 160 °C. The rubber sample was subsequently cooled down to 60 °C (cooling rate of 30 °C/min), followed by temperature stabilization for 3 min. Finally, the sample was deformed from 0.1% to 200% at frequency *f* = 1 Hz. Again, three measurements were performed for each compound.

#### 2.3.4. The Hysteresis Characteristics

The hysteresis characteristics of the rubber compounds were evaluated on the ZwickRoell tensile testing machine (ZwickRoell, Ulm, Germany) in compression mode. The samples measuring 100 mm in diameter and 20 mm in thickness were tested. Cyclic deformation of 10% at 50 mm/min was applied to each sample. The total number of 100 cycles was performed to reach a steady state of the hysteresis loop. The tests were carried out at 23 °C. Cycling loading tests of vibration damping were also performed with the same machine. The rubber samples were cyclically loaded for 0; 100,000; 250,000; 500,000; and 750,000 cycles at a constant force of 10 kN, excitation frequency *f* = 20 Hz, and ambient temperature of 23 °C.

#### 2.3.5. Mechanical Vibration Damping Measurement

In general, vibration measurement methods are divided into contacting and contactless types [17,18,19,20]. The contactless vibration measurement methods can be performed using inductive, capacitive, optical (e.g., laser triangulation and laser interference), and ultrasonic sensors. They can be applied to measure deflections of rotary components, as well as higher vibration amplitudes and excitation frequencies compared to the contacting vibration measurements, which can be performed using piezoelectric and microelectro mechanical system (MEMS) accelerometers. The accelerometers are mounted directly on the vibrating component and are being used more widely due to the rapid development of electronic technology, accompanying secondary instrument, and low-noise cables, and their high insulation resistance and small capacitance.

The vibration damping properties of the investigated rubber composites can be examined by different methods, namely using the free vibration method and by using harmonically excited vibration [21,22]. The free vibration method evaluates a material´s ability to dampen mechanical vibrations based on the logarithmic decrement *δ* and the damping ratio *ζ*. For harmonically excited vibration, which can be achieved under harmonic force or under the harmonic motion of a base, the vibration damping properties are characterized by the frequency dependencies of amplitude ratios (e.g., amplification factor or displacement transmissibility). The method involving harmonically excited vibration based on the response of a damped system under the harmonic motion of the base (so-called kinematic excitation), which is relatively simple, was applied in order to investigate the vibration damping properties of the studied rubber composites.

A material´s ability to damp mechanical vibration can be characterized by the transfer damping function *D* (dB), which is expressed by the following equation [23]:

(1) $$D=20\cdot \log\frac{v_{01}}{v_{02}}$$

where *v*_01_ is the velocity amplitude on the input (i.e., excitation) side of the tested sample and *v*_02_ is the velocity amplitude on the output side of the tested sample. For harmonically excited vibration, it is also possible to express the transfer damping function as follows:

(2) $$D=20\cdot \log\frac{y_{01}}{y_{02}}=20\cdot \log\frac{a_{01}}{a_{02}}$$

where *y*_01_ (*a*_01_) is the displacement (acceleration) amplitude on the input side of the tested sample and *y*_02_ (*a*_02_) is the displacement (acceleration) amplitude on the output side of the tested sample. There are three different types of mechanical vibration depending on the transfer damping function value, namely damped (*D* > 0), undamped (*D* = 0), and resonance (*D* < 0) vibration.

**Figure 1:**
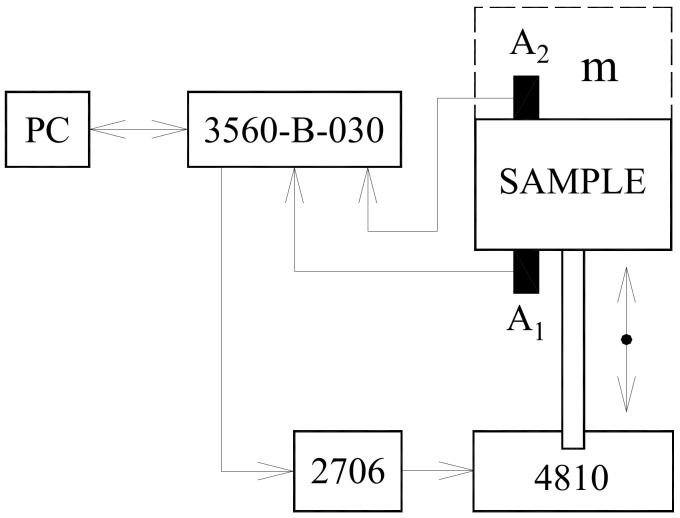
Schematic diagram of the measuring device.

**Figure 2:**
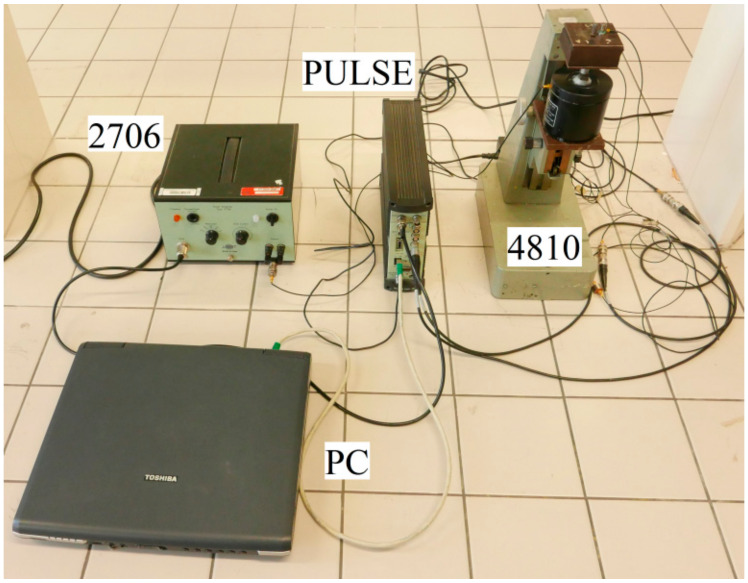
View of the experimental setup used for the vibration damping testing. Legend of the abbreviations: PC—personal computer; PULSE-signal pulse multi-analyzer; 2706—power amplifier; 4810—mini-shaker.

The mechanical vibration damping testing of the tested rubber composite materials was performed using the forced oscillation method. The transfer damping function was experimentally measured using the BK 4810 mini-shaker (Brüel and Kjær, Nærum, Denmark) in combination with a BK 3560-B-030 signal pulse multi-analyzer (Brüel and Kjær, Nærum, Denmark) and a BK 2706 power amplifier (Brüel and Kjær, Nærum, Denmark) at the frequency range of 2–3200 Hz (see Figure 1). Sine waves were generated by the mini-shaker. The acceleration amplitudes on the input and output sides of the investigated specimens were recorded by the BK 4393 A_1_ and A_2_ piezoelectric accelerometers (Brüel and Kjær, Nærum, Denmark). The accelerometers have these parameters: the frequency ranges from 0.5 to 16,500 Hz; the temperature range from −74 to 250 °C; and the weight is 2.4 g [24]. Measurements of the transfer damping function were performed for different inertial masses *m* (i.e., for 0, 90 and 500 g), which were located on the upper side of the harmonically loaded investigated samples (see Figure 1). Moreover, vibration damping properties of the investigated rubber samples with ground plane dimensions of 60 mm × 60 mm were performed for three different thicknesses (i.e., for 10, 15, and 20 mm) of these materials. The view of the experimental setup used for the vibration damping testing is shown in Figure 2. Each measurement was repeated 10 times at an ambient temperature of 22 °C.

## 3. Results and Discussion

### 3.1. Curing Characteristics

The curing characteristics of the mixed compounds are given in Table 2. It is evident that the minimum torque *M_L_* significantly decreased with an increase in the primary carbon black particle size. This is caused by the decreasing amount of the immobilized rubber chains on the decreasing carbon black surface, as well as the structure. The low aggregate structure and surface area of the N 990 type leads to weak interaction with rubber chains. Moreover, the thermal production process leads to a higher purity of the carbon black surface, with low content of active groups. This fact can result in a lower stiffening effect. However, in the case of the torque *M_L_*, interactions between rubber chains and carbon black are mainly caused by physical forces, because the property is in an uncured state [25,26,27,28].

The difference between the maximum (*M_H_*) and the minimum (*M_L_*) torques is marked as Δ*M*, which is a parameter demonstrating the degree of chemical crosslinking. The same amount and type of curing system was observed in the compounds; thus, the degree of Δ*M* is supposed to be comparable for all compounds. In reality, the Δ*M* is significantly different. Evidently, the reaction between the rubber and the curing system is one of the factors affecting the crosslinking process [28,29].

The other factor affecting the crosslinking process is the chemical reaction of the rubber with functional groups on the carbon black surface, which differs depending on the carbon black grade. It was found that these functional groups could have either positive or negative effects on the curing characteristics, depending on carbon black type [29]. Although the N 330 type has a higher surface area compared to the N 550 type, its curing level is lower due to the presence of cure-retarding groups [30]. This phenomenon causes various *t*_90_ values for the compounds in this study.

### 3.2. Quasi-Static Test Results

The parameters of mechanical properties of the SBR and carbon black compounds are presented in Table 3. The hardness testing is the most obvious mechanical test. In this case, the Shore A hardness was measured. The obtained hardness results decreased from 69 to 55 Shore A and corresponded to the carbon black particle size, ranging from the largest specific surface (N 110) to the smallest one (N 990). The higher specific surface led to a harder rubber compound. It is evident from the above results that the hardness generally decreased with a decrease in the carbon black surface area.

The tensile test was performed in order to characterize the basic mechanical properties of the rubber compounds. It should be noted that the compounds containing the carbon black with small primary particles (N 110 and N 330) showed the highest break stress (about 23 MPa), while the elongation was the lowest. The increasing primary carbon black particle size causes the decrease of the break stress. This is caused by the reinforcing ability of the carbon black particle size. The smaller primary particle sizes led to a higher specific surface area and to a stronger restriction of the elastomer chain mobility.

Here, the 300% modulus is the parameter characterizing the stiffness of rubber vulcanizates, representing the stress at 300% extension. In addition to previous parameters, it is influenced by the carbon black surface area. The highest value for this modulus was obtained for the N 110 type. The 300% modulus decreased with a decrease in the surface area as well.

The rebound resilience increased from 38% up to 51% for N 110 and N 990 carbon black types, respectively. Additionally, this property is influenced by the particle size, and thus, by the surface area of the carbon black. The stiffening effect of the N 110 type is stronger in comparison to the others. It provides harder rubber with lower rebound elasticity, because a larger part of the mechanical energy is transformed into heat.

Theoretically, the mechanical properties should show greater differences between the N 110 and N 330 carbon black types. Unfortunately, mechanical properties are strongly influenced by the level of filler dispersion in the matrix, which also depends on the carbon black particle size. With decreasing particle size, more energy is required to achieve high filler dispersion. Thus, a shorter mixing time leads to poorer filler dispersion. For this reason, the mechanical properties are reduced. On the other hand, a longer mixing time can cause polymer chain scission and also a decrease in the final properties. For this study, the rubber compounds were prepared under constant mixing conditions.

### 3.3. Dynamical Mechanical Properties

**Figure 3:**
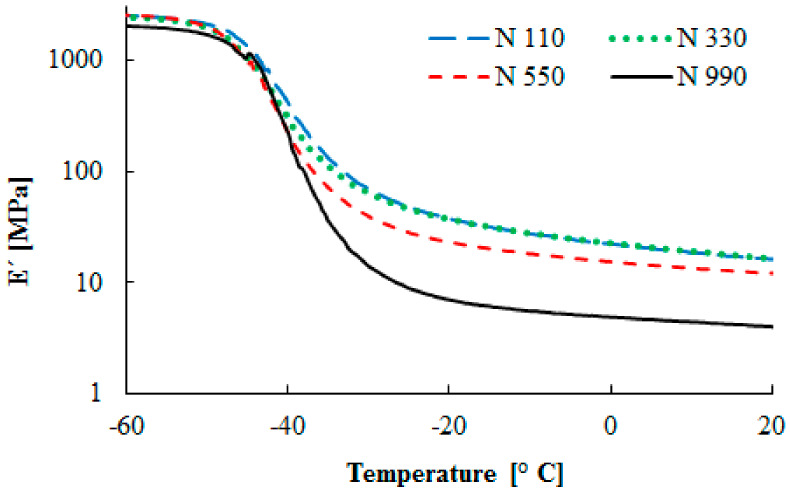
Temperature dependence of the tensile storage modulus.

The effects of various carbon blacks in the SBR matrix on the temperature dependence of the elastic (storage) modulus is summarized in Figure 3. These curves describe the temperature region, where the hard and brittle material behavior is replaced by the rubbery or viscoelastic behavior. The modulus decreased strongly in the glassy transition region. The elastomer chain mobility, and thereby the glass temperature, is noticeably affected by various additives in the rubber compound. The presence of filler, the amount, an increase in the surface area, or the structure can restrict the chain’s movement ability and lead to the shift of the glassy region to higher or lower temperatures [9].

During heating, the elastic modulus of the studied compounds decreased in the temperature range from −50 °C to −25 °C. The most rapid decrease of the elastic modulus was observed for the compound containing the N 990 carbon black type. On the other hand, the highest values of the elastic modulus were obtained for the compounds with the N 110 and N 330 carbon black types. This is connected with the decreasing carbon black particle sizes and the increasing surface area.

**Figure 4:**
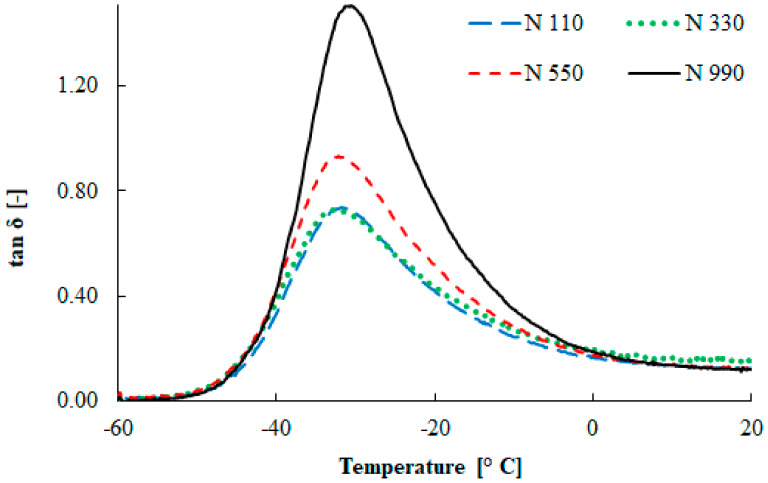
Temperature dependence of the loss factor.

The glass transition temperature for the rubber compounds determined from the loss factor (*tan δ*) curve of three measurements was in the low temperature region, namely −31.9 ± 0.6 °C, −32.5 ± 0.5 °C, −32.2 ± 0.6 °C, and 31.1 ± 0.8 °C for the N 110, N 330, N 550, and N 990 types, respectively (see Figure 4). The differences in the glass temperature between compounds were less than 10%. The shift in glass transition temperature was probably caused by sample thickness inhomogeneity and the slightly different clamping forces of each compound. As a result, no significant effect was observed for the particle size on the glass transition temperature. In addition, the damping properties of the rubber compounds can be evaluated from this figure. It is evident that the loss factor increased with an increase in the primary particle size.

**Figure 5:**
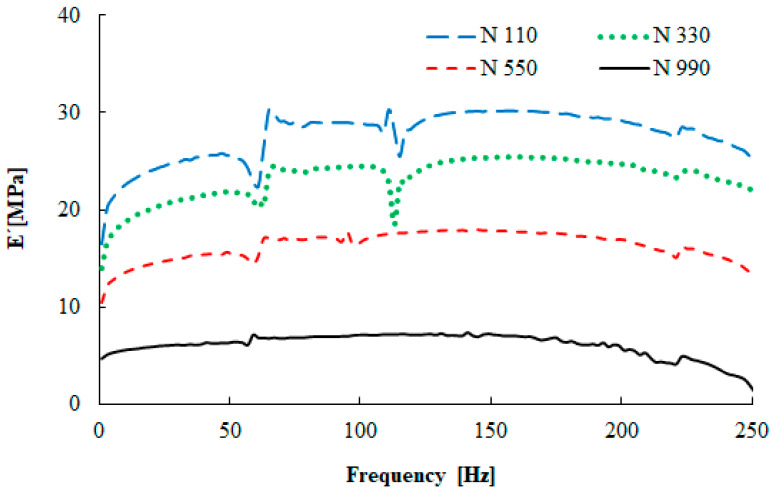
Frequency dependence of the tensile storage modulus (displacement x = 5 μm, T = 25 °C).

**Figure 6:**
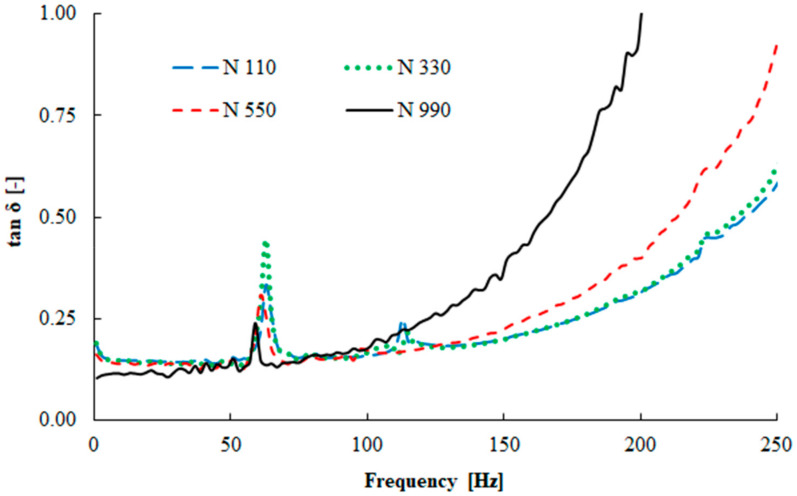
Frequency dependence of the loss factor (displacement x = 5 μm, T = 25 °C).

The dynamic stiffness of the tested rubber mixtures is measured by the complex modulus of elasticity. The frequency dependencies of the storage modulus and the loss factor at 25 °C are demonstrated in Figure 5 and Figure 6.

Significant changes of the storage modulus depending on the frequency for SBR compounds with the N 110, N 330, and N 550 carbon blacks are evident in Figure 5. There is a moderate increase of the storage modulus with the frequency up to 180 Hz. The filler with the smallest primary particle size (N 110) exhibited the highest storage modulus values, while N 990 gave the lowest values. This is connected with the filler–polymer interaction. This fact depends on the filler’s primary particle size, as well as its structure [28].

It was found that the loss factor *tan δ* at low frequencies up to 100 Hz showed similar behavior for all carbon black compounds (see Figure 6). Generally, the loss factor increased with an increase in the frequency. While for the more reinforced carbon black type (N 110) the increase of *tan δ* was slower, in the case of the N 990 type the loss factor increased more significantly.

**Figure 7:**
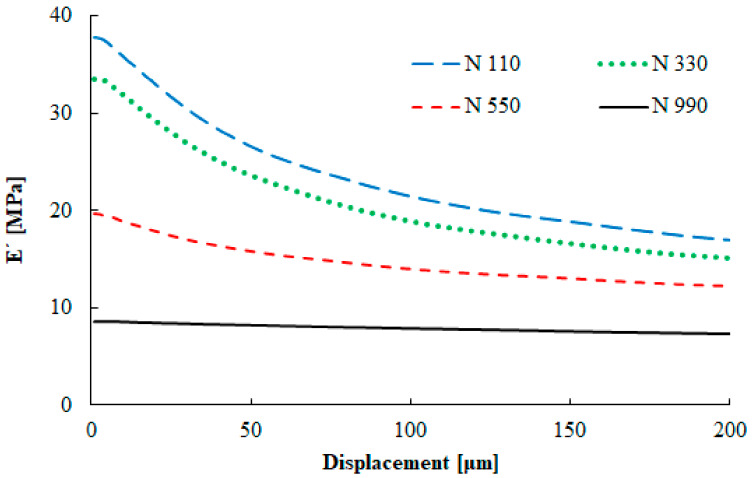
Displacement dependence of the tensile storage modulus (f = 20 Hz, T = 0 °C).

The next measurement was performed in order to evaluate the deformation dependence of the tested samples on their dynamic properties. The elastic modulus dependence on the displacement is shown in Figure 7. The effects of the carbon black particle size and structure were measured by this experiment. The compound with the smallest primary particle size gave the highest elastic modulus value, while that with the largest particle sizes showed almost no stiffening effect and had almost constant elastic modulus during the whole deformation range. High elastic moduli at low deformation levels are caused by the filler–filler interaction, as explained by Payne [31]. The smallest particles with higher carbon black structures created stronger filler–filler network in comparison with larger particles and a lower carbon black structures. With the increasing deformation, the filler network is destroyed and the interaction between the rubber and carbon black becomes the main factor.

Incorporated carbon black particles, which are already known as aggregates, create a filler–filler network. Inside the rubber compound, carbon black aggregates are formed by van der Waals forces into so called agglomerates. If a small deformation is applied, a high elastic modulus value is obtained.

The reason for this phenomenon is the strong interaction between filler particles that are not broken. From Payne’s point of view, some of the rubber is immobilized on the filler surface, and in addition some of the rubber is also immobilized inside the branched structure of the agglomerate (known as occluded rubber). If the deformation increases, the agglomerates are broken into smaller sizes, and therefore the elastic modulus decreases. This phenomenon is caused by more mobile smaller units inside the rubber compound. At high deformation levels a plateau can be reached, whereby individual mobile aggregates units are set into motion. This behavior is known as the Payne effect [31,32,33,34,35,36].

**Figure 8:**
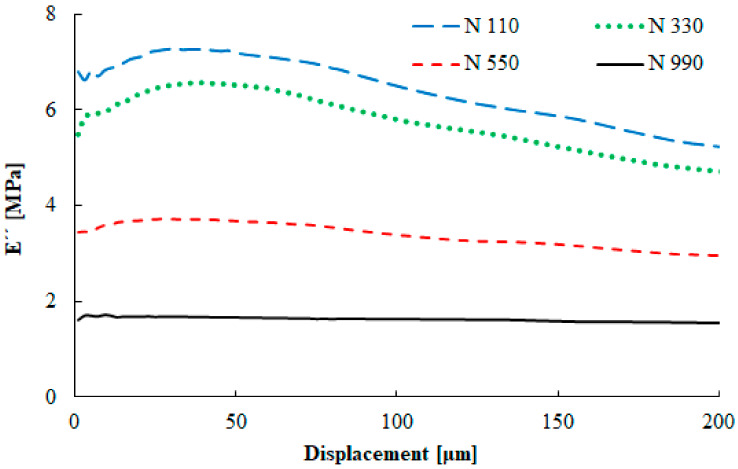
Displacement dependence of the tensile loss modulus (f = 20 Hz, T = 0 °C).

The dependence of the loss modulus on the sample deformation is presented in Figure 8. The loss modulus increased with a decrease in the primary particle size. The stronger filler–filler interaction led to higher energy dissipation. With the increasing deformation, the loss modulus maximum was found around the displacement amplitude of 35 µm for the compounds with the N 110 and N 330 carbon black types. The highest damping properties were obtained in this area. Apparently, the values of the loss modulus were low for the N 550 and N 990 carbon black types, while damping properties were especially negligible for the N 990 type.

Shear amplitude deformation was measured using a rubber process analyzer. This test describes similar behavior of the rubber compounds as the DMA measurements, but in shear deformation mode.

**Figure 9:**
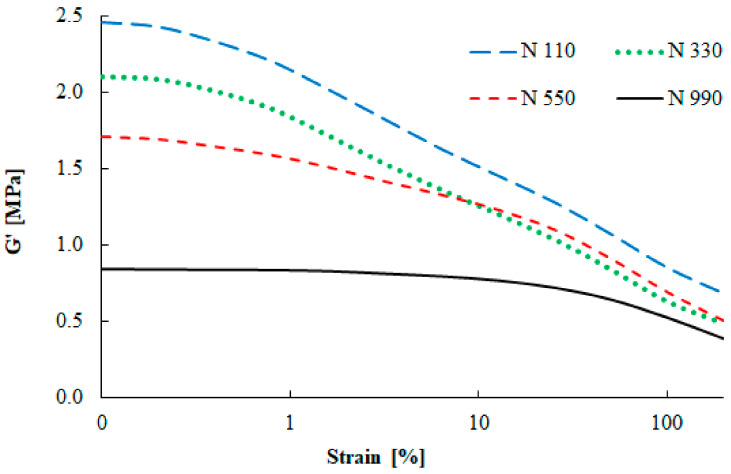
Strain amplitude dependence of the shear storage modulus.

**Figure 10:**
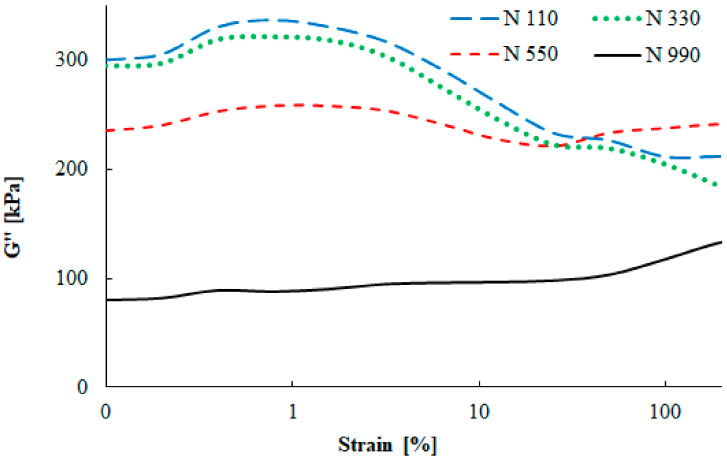
Strain amplitude dependence of the shear loss modulus.

The dependence of the filler–filler interaction for compounds with the N 110, N 330, N 550, and N 990 carbon black types is depicted in Figure 9. A strong dependence of the shear storage modulus on the filler particle size is visible, similarly to the DMA testing. Large carbon black particles (N 990) are characterized by a low reinforcing effect, while small particles (N 110) cause a pronounced increase of the storage modulus. Figure 10 shows the strain amplitude dependence of the loss modulus of four carbon black grades. Similarly to the DMA measurements, the shear loss modulus was highest for the N 110 type, while for the N 990 type it was the lowest. The maximum value of the loss modulus was observed at 1% strain, which is fully connected with the maximal damping properties.

### 3.4. The Hysteresis Characteristics

Due to the viscoelastic nature of the rubber vulcanizates, the stress–strain curves of the tested rubber create a hysteresis loop during the loading and unloading cycles. The hysteresis loop area corresponds to the energy dissipated into heat. The heat generation inside the rubber mixture can lead to it softening and even rupturing. The heat generation is affected by the polymer nature, curing level, and compound composition. This behavior is also known as the Mullins effect.

**Figure 11:**
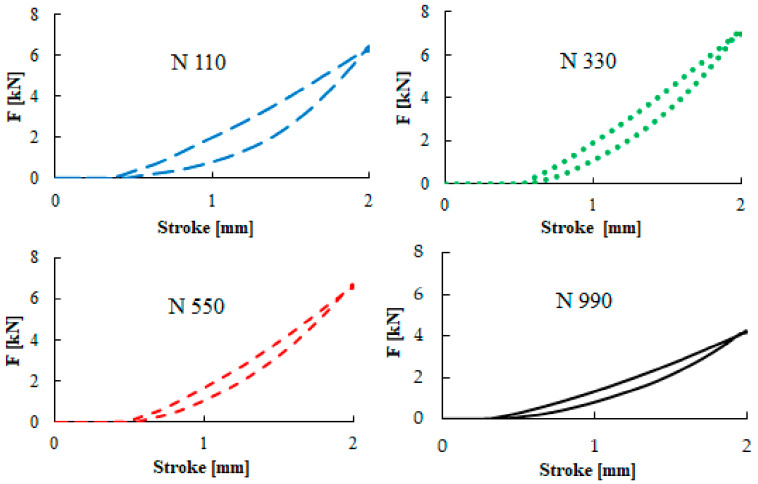
Hysteresis images of four carbon black types.

The hysteresis loops for the studied compounds are presented in Figure 11. The rubber was dynamically loaded in compression mode during 100 loading cycles. In this figure, the last cycle is recorded. Evidently, there are quite large differences among the carbon black types added to each compound. The largest area of the hysteresis loop was achieved for the N 110 carbon black type, which had the highest specific surface area, while the lowest heat generation was obtained for the N 990 type. An explanation of this phenomena was given by Fukahori [36]. According to this theory, rubber covers the carbon black surface in creation of the so-called bound rubber. This is an immobilized part of the rubber macromolecules that is physically connected with carbon black particles. During the loading of a polymer–carbon black structure, the orientation of this structure appears. If the unloading process is applied, the stress decreases faster compared to common macromolecular stress relaxation, and the decrease in the unloading curve is visible.

### 3.5. Vibration Damping Properties

**Figure 12:**
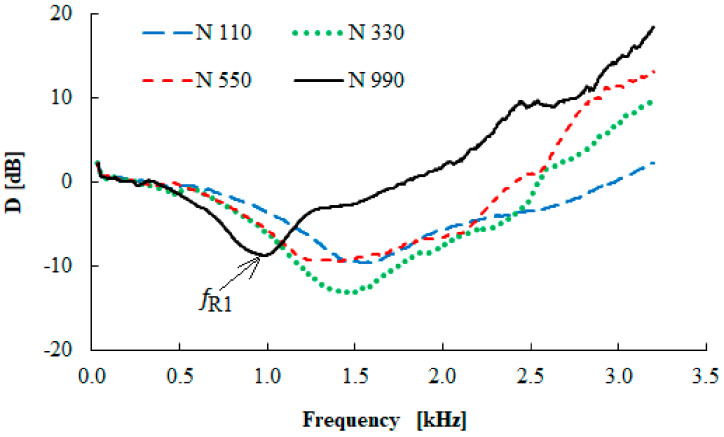
Frequency dependence of the transfer damping function (D) for the tested rubber composite measuring t = 10 mm in thickness, without inertial mass (m = 0 g).

Examples of the frequency dependencies of the transfer damping function of the tested rubber composites measuring *t* = 10 mm in thickness with different carbon black particle sizes are shown in Figure 12. It is evident from this comparison that the size of the carbon black particles has a significant influence on the vibration damping properties. It can be concluded that the material´s ability to damp mechanical vibration generally increased with an increase in the carbon black particle size. This is caused by lower stiffness (or by higher damping) of the rubber composites, which were produced with larger carbon black particle sizes. These facts result in a higher transformation of input mechanical energy into heat during forced oscillations [37] and a decrease in the values of the damped and undamped natural frequencies [38]. Therefore, the first resonance frequency (*f_R_*_1_) value was shifted to the left (see Figure 12) with an increase in the carbon black particle size, i.e., from 1520 (N 110) to 968 Hz (N 990), as indicated in Table 4. These findings are in excellent agreement with the results that were experimentally determined by the abovementioned methods, namely the hardness, tensile, shear, and viscoelastic measurements. It was verified in these cases that the increasing carbon black particle size led to a decrease of the break stress, the Shore A hardness, and the storage moduli *E´* and *G´*. In contrast, the rebound resilience was higher for these particle sizes.

**Figure 13:**
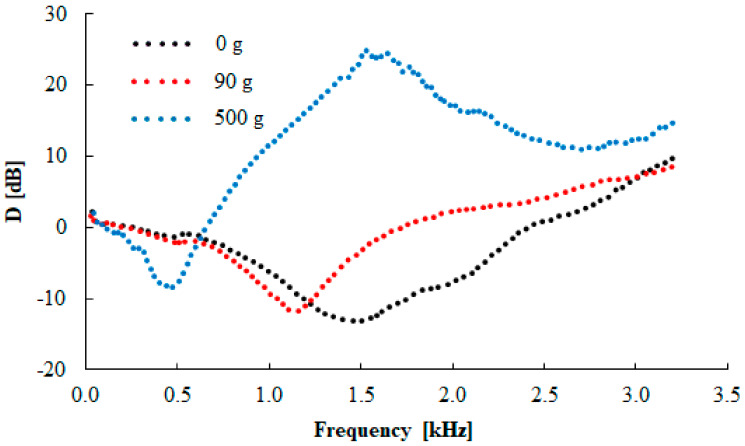
Frequency dependencies of the transfer damping function (D) for the tested N 330 rubber composite measuring t = 10 mm in thickness, loaded with different inertial masses.

**Figure 14:**
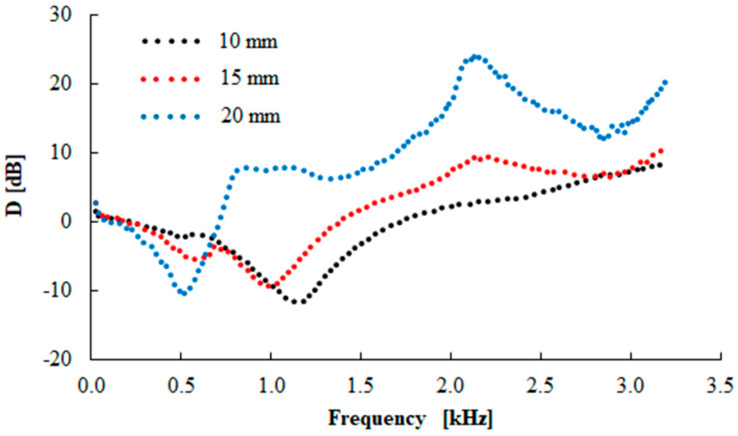
Frequency dependencies of the transfer damping function (D) for the tested N 330 rubber composites of different thicknesses and loaded with inertial mass m = 90 g.

The vibration damping properties of the investigated harmonically loaded composite rubber samples are also influenced by their thickness *t*, the excitation frequency *f*, and the inertial mass *m*. The effect of the inertial mass on the vibration damping properties of sample N 330 is shown in Figure 13. It is visible that better damping properties were obtained with higher inertial mass *m*, which led to a decrease in the undamped natural frequency, and thus, the damped frequency. This is due to the fact that the natural frequency of an undamped system is proportional to the square root of the material stiffness for the applied inertial mass [22]. For this reason, the inertial mass has a positive influence on vibration damping, which is reflected by a shift of the first resonance frequency peak position to lower frequencies, i.e., by the decrease of the *f_R_*_1_ (see Table 4) from 1441 (*m* = 0 g) to 412 Hz (*m* = 500 g). The vibration isolation properties of the investigated rubber composites are also significantly influenced by their thickness *t*, as shown in Figure 14 for the N 330 sample with an inertial mass *m* of 90 g. It is evident that the higher material thickness led to lower values of the *f_R_*_1_, i.e., from 1138 (*t* = 10 mm) to 520 Hz (*t* = 20 mm), as indicated in Table 4. For this reason, the rubber thickness generally has a positive influence on vibration damping properties. It is also visible from Figure 12, Figure 13 and Figure 14 that the material´s ability to damp mechanical vibration is significantly influenced by the excitation frequency *f*. It is evident that resonant mechanical vibration (*D* < 0) was achieved at low excitation frequencies, depending on the rubber sample type, the thickness *t*, and the inertial mass *m*. For example, for the N 110 sample type measuring *t* = 10 mm in thickness and without inertial mass (*m* = 0 g), the resonant mechanical vibration was observed at frequencies *f* < 2950 Hz (see Figure 12). For the N 990 sample type measuring *t* = 20 mm in thickness and with inertial mass *m* = 500 g, the resonant mechanical vibration was achieved at considerably lower excitation frequencies (at *f* < 330 Hz). In contrast, damped mechanical vibration (*D* > 0) was generally achieved at higher excitation frequencies (see Figure 12, Figure 13 and Figure 14).

**Figure 15:**
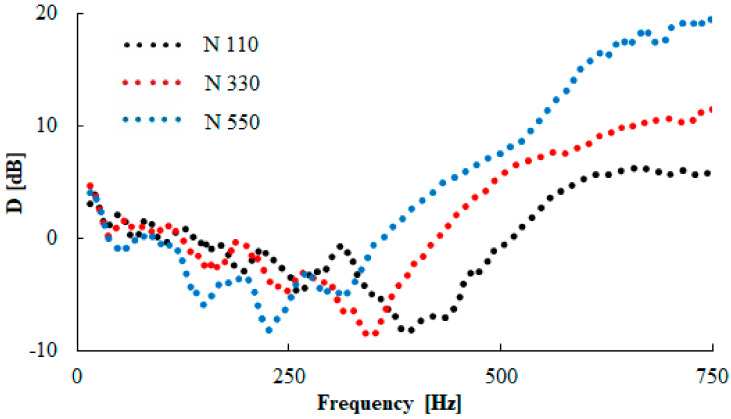
Frequency dependencies of the transfer damping function (D) for the tested rubber composites measuring t = 20 mm in thickness after 750,000 loading cycles, with inertial mass m = 90 g.

The material´s ability to damp mechanical vibration was also evaluated for the tested material samples, which were harmonically loaded by the compression force with an amplitude of 10 kN at an excitation frequency of 20 Hz. In the case of the N 990 sample type, it was not possible to perform this evaluation due to the low stiffness of this rubber sample compared to the other tested rubber sample types. The frequency dependencies of the transfer damping function of the investigated rubber samples (thickness *t* = 20 mm, inertial mass *m* = 90 g) after 750,000 loading cycles are demonstrated in Figure 15. Again, as in the case of the cyclically unloaded rubber samples (see Figure 12), the material´s ability to dampen mechanical vibration increased with an increase in the carbon black particle size. For this reason the rubber composites, which were produced with larger carbon black particle sizes, exhibited lower stiffness, resulting in a decrease of the first resonance frequency peak position to lower excitation frequencies. As shown in Table 5, similar results were obtained independently of the number of loading cycles.

**Figure 16:**
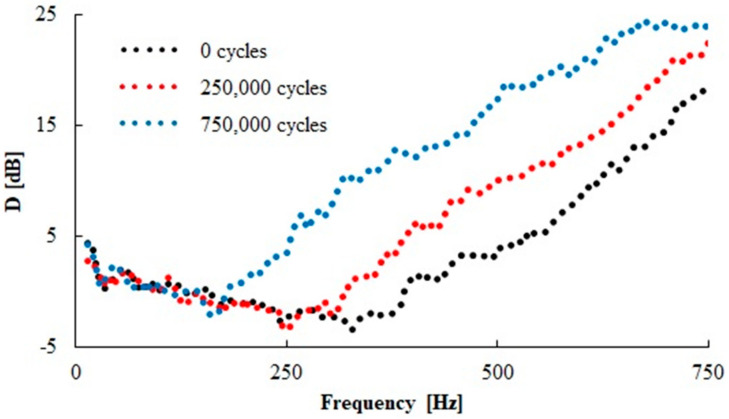
Effect of number of loading cycles on frequency dependencies of the transfer damping function (D) for the N 330 rubber composite measuring t = 20 mm in thickness, with inertial mass m = 500 g.

The effect of the number of loading cycles on the vibration damping properties of the N 330 sample type is shown in Figure 16. It is evident that the vibration damping ability of the sample generally increased with an increase in the number of loading cycles, which led to a decrease of the first resonance frequency peak position to lower frequency values (see Table 5) with the increasing number of loading cycles. Therefore, the higher number of loading cycles led to a reduction in rubber sample stiffness, which was accompanied by better damping properties in this rubber sample. As shown in Table 5, similar findings were observed for the other tested rubber composites.

## 4. Conclusions

Mechanical vibrations are currently undesirable in many cases. Therefore, this vibration must be eliminated in appropriate ways. One of the possible elimination methods is the application of suitable vibro-insulating materials. This paper was focused on the study of the mechanical and vibro-isolation properties of rubber compounds containing different carbon black particle sizes. Nevertheless, their volume concentration was the same. On the basis of the evaluated measurements, it can be stated that the particle size of the carbon black in the rubber composites had a significant effect on the stiffness of the rubber, and thus on its mechanical and vibro-isolation properties.

The mechanical properties of the tested rubber mixtures were investigated for reflective elasticity, tensile, viscoelastic, and vibro-insulating properties. It was found in this work that the stiffness of the rubber samples generally decreased with an increase in the particle size, resulting in higher reflective elasticity and lower mechanical properties, including lower hardness (decrease from 69 to 55 Sh A), breaking stress, and real components of the complex modulus of elasticity (tensile and shear). On the other hand, the higher particle size in the rubber mixtures led to a greater loss factor and deformations in terms of sample breakage.

The above facts were in good agreement with the vibro-isolation tests for the observed rubber materials, which were examined using the forced oscillation method based on the transfer damping function. At the same time, a material´s ability to dampen mechanical vibrations under dynamic stress is associated with the first resonance frequency, which is generally lower for materials that better dampen mechanical oscillation (or for materials with lower stiffness). Based on this method, it was verified that the first resonant frequency generally decreased with an increase in the carbon black particle size. Therefore, larger carbon black particles of the same volume concentration in rubber patterns contributed to better damping of mechanical vibrations, resulting in a higher transformation of input mechanical energy into heat under dynamic loading of these rubber composite samples. Vibration damping properties were also evaluated for the investigated rubber samples, which were harmonically loaded by a compression force. It can be concluded that a higher number of loading cycles led to a stiffness reduction in the investigated rubber composites, which was accompanied by a shift of the first resonance frequency peak position to lower excitation frequencies. Depending on the rubber type and the inertial mass, the decrease of the first resonance frequency after 750,000 loading cycles was between 37% and 65%. Furthermore, it has been found in this work that the material´s ability to damp mechanical vibrations generally increased with an increase in the excitation frequency of mechanical vibration, inertial mass, and thickness of the investigated rubber samples.

## Figures and Tables

**Table 1:** Rubber compound recipe. SBR, styrene butadiene rubber; TBBS, N-tert-butyl-benzothiazole sulfonamide.

Ingredients	Loading [phr]
SBR	100
Carbon black	50
ZnO	3
Stearic acid	1
TBBS	1
Sulfur	1.75

**Table 2:** Curing characteristics of the studied compounds.

Rubber Type	*M_L_* [N·m]	*M_H_* [N·m]	Δ*M* [N·m]	*t_s_*_1_ [min]	*t*_90_ [min]
N 110	0.27	1.71	1.44	2.6	10.5
N 330	0.24	1.45	1.21	2.6	11.5
N 550	0.23	1.51	1.28	3.0	12.8
N 990	0.12	1.07	0.95	3.3	12.6

**Table 3:** Mechanical properties of the rubber compounds.

Rubber Type	Shore A [Sh A]	Stress at Break [MPa]	Strain at Break [%]	300% Modulus [MPa]	Resilience [%]
N 110	69 ± 1	23.0 ± 1.4	480 ± 36	13.0 ± 1.3	38 ± 1
N 330	66 ± 1	23.9 ± 0.9	520 ± 23	12.7 ± 1.0	39 ± 1
N 550	64 ± 1	19.8 ± 0.5	510 ± 25	11.9 ± 0.5	44 ± 1
N 990	55 ± 1	11.9 ± 2.7	640 ± 75	4.1 ± 0.4	51 ± 1

**Table 4:** The first resonance frequency (f_R1_) in Hz of the studied rubber composite materials, as induced by harmonic force vibration for different rubber thicknesses and inertial masses.

Rubber Type	Thickness [mm]	Inertial Mass [g]		
		0	90	500
N 110	10	1520 ± 41	1146 ± 18	430 ± 15
	15	1293 ± 29	1043 ± 18	419 ± 14
	20	1080 ± 15	928 ± 17	354 ± 12
N 330	10	1441 ± 32	1138 ± 19	412 ± 14
	15	1242 ± 22	957 ± 16	402 ± 13
	20	881 ± 16	520 ± 15	320 ± 9
N 550	10	1404 ± 27	900 ± 18	379 ± 13
	15	977 ± 18	737 ± 16	288 ± 10
	20	718 ± 16	456 ± 12	233 ± 8
N 990	10	968 ± 20	848 ± 16	298 ± 10
	15	802 ± 19	635 ± 15	258 ± 9
	20	693 ± 15	310 ± 11	231 ± 7

**Table 5:** The first resonance frequency (f_R1_) in Hz of the studied rubber composite materials measuring t = 20 mm in thickness, as induced by harmonic force vibration for different inertial masses and numbers of loading cycles.

Number of Cycles	Rubber Type	Inertial Mass [g]		
		0	90	500
0	N 110	1080 ± 15	928 ± 17	354 ± 12
	N 330	881 ± 16	520 ± 15	320 ± 9
	N 550	718 ± 16	456 ± 12	233 ± 8
100,000	N 110	1029 ± 17	563 ± 14	328 ± 11
	N 330	534 ± 15	507 ± 13	301 ± 10
	N 550	430 ± 13	378 ± 11	227 ± 9
250,000	N 110	482 ± 15	430 ± 12	245 ± 10
	N 330	457 ± 12	405 ± 11	241 ± 9
	N 550	381 ± 10	356 ± 10	192 ± 8
500,000	N 110	434 ± 11	388 ± 10	237 ± 8
	N 330	399 ± 11	371 ± 11	231 ± 8
	N 550	372 ± 10	237 ± 9	140 ± 6
750,000	N 110	404 ± 12	380 ± 11	208 ± 8
	N 330	374 ± 11	338 ± 10	158 ± 7
	N 550	360 ± 11	214 ± 8	126 ± 5
